# 520. From Night to Day: Capturing Sleep and Pain in Hidradenitis Suppurativa Using Daily Diaries

**DOI:** 10.1093/jbcr/irag033.217

**Published:** 2026-04-06

**Authors:** Lynn Nakad, Sheera F Lerman, Julie A Caffrey, Carrie A Cox, Jim Stone, Phoebe Wang

**Affiliations:** Johns Hopkins University, Baltimore, MD; Johns Hopkins University School of Medicine, Baltimore, MD; Johns Hopkins University School of Medicine, Baltimore, MD; Johns Hopkins Burn Center, Baltimore, MD; Johns Hopkins University Bayview Medical Center, Baltimore, MD; Johns Hopkins University, Westminster, MD

## Abstract

**Introduction:**

Hidradenitis suppurativa (HS) is a painful, inflammatory skin disease often requiring surgical excision in advanced stages. Sleep regulates immune function and sleep disturbances are a risk factor for chronic pain, yet little is known about sleep in HS. This study characterized sleep in HS and examined its relationship with pain and quality of life (QoL).

**Methods:**

HS patients scheduled for surgical excision of HS lesions completed baseline assessments on pain, sleep, and QoL. Patients completed daily sleep diaries for 7 days from which the following sleep continuity variables were calculated: total sleep time (TST), sleep onset latency (SOL), wake after sleep onset (WASO), and sleep efficiency (SE). Pain intensity, interference, and catastrophizing were assessed with the Brief Pain Inventory short form (BPI) and Pain Catastrophizing Scale (PCS). QoL was measured with the Dermatology Life Quality index (DLQI). Associations between sleep, pain, and QoL were evaluated.

**Results:**

22 participants (mean age = 36.8; 84% female) reported moderate pain severity and interference (BPI severity = 5.7; interference = 2.9), high catastrophizing (PCS = 35.1), and impaired QoL (DLQI = 13.7). Mean insomnia severity index (ISI) score was 12.5 and Epworth Sleepiness Scale was 7.3. Sleep diaries demonstrated mean TST of 6.9 hrs, SOL of 34.9 min, WASO of 34.9 min, and median SE of 82.7%. Bivariate analyses showed greater pain severity correlated with shorter TST (r = -0.66, *p*<.001), lower SE (r = -0.57, *p*=.005), and longer WASO (r = 0.43, *p*=.045). Pain interference correlated with lower SE (r = -0.49, *p*=.022) and greater WASO (r = 0.47, *p*=.028). QoL was not significantly correlated with sleep variables. Pain catastrophizing correlated with longer SOL (r = 0.52, *p*=.013), greater WASO (r = 0.55, *p*=.008), and lower SE (r = -0.48, *p*=.023). In adjusted regression models controlling for age, sex, ISI, and PCS, shorter TST was significantly associated with greater pain severity (β = -0.016, 95% CI [-0.029, -0.002], *p*=.025).

**Conclusions:**

This is the first study to capture daily patterns of sleep in patients with HS using daily diaries. HS patients demonstrated increased sleep disturbances. Additionally, pain severity was associated with reduced sleep duration and efficiency, and pain catastrophizing contributed to delayed sleep onset and fragmented sleep. After adjusting for covariates, shorter sleep duration was a robust predictor of pain severity. These findings highlight sleep as a critical treatment target that may hold promise for improving pain outcomes in HS.

**Applicability of Research to Practice:**

Addressing sleep disturbances with behavioral or pharmacological interventions alongside standard care may provide an effective adjunct strategy to improve pain management in HS.

**Funding for the study:**

N/A.

